# Human papillomavirus-associated head and neck squamous cell carcinoma cells lose viability during triggered myocyte lineage differentiation

**DOI:** 10.1038/s41419-024-06867-4

**Published:** 2024-07-19

**Authors:** Sarah Gendreizig, Laura Martínez-Ruiz, Alba López-Rodríguez, Harkiren Pabla, Leonie Hose, Frank Brasch, Tobias Busche, Germaine Escames, Holger Sudhoff, Lars Uwe Scholtz, Ingo Todt, Felix Oppel

**Affiliations:** 1 Department of Otolaryngology, Head and Neck Surgery, Campus Klinikum Bielefeld Mitte, University Hospital OWL of Bielefeld University, Teutoburger Str. 50, 33604 Bielefeld, Germany; 2 Institute of Biotechnology, Biomedical Research Center, Health Sciences Technology Park, University of Granada, Granada, Spain; 3 Department of Physiology, Faculty of Medicine, University of Granada, 18016 Granada, Spain; 4 Centro de Investigación Biomédica en Red Fragilidad y Envejecimiento Saludable (CIBERFES), Instituto de Investigación Biosanitaria (Ibs), Granada, San Cecilio University Hospital, Granada, Spain; 5 Department of Pathology, Klinikum Bielefeld, Teutoburger Str. 50, 33604 Bielefeld, Germany; 6 Center for Biotechnology (CeBiTec), University Hospital OWL of Bielefeld University, Bielefeld, Germany

**Keywords:** Oral cancer, Mechanisms of disease, Cell death

## Abstract

Head and neck squamous cell carcinoma (HNSCC) is a highly malignant disease, and death rates have remained at approximately 50% for decades. New tumor-targeting strategies are desperately needed, and a previous report indicated the triggered differentiation of HPV-negative HNSCC cells to confer therapeutic benefits. Using patient-derived tumor cells, we created a similar HNSCC differentiation model of HPV+ tumor cells from two patients. We observed a loss of malignant characteristics in differentiating cell culture conditions, including irregularly enlarged cell morphology, cell cycle arrest with downregulation of Ki67, and reduced cell viability. RNA-Seq showed myocyte-like differentiation with upregulation of markers of myofibril assembly. Immunofluorescence staining of differentiated and undifferentiated primary HPV+ HNSCC cells confirmed an upregulation of these markers and the formation of parallel actin fibers reminiscent of myoblast-lineage cells. Moreover, immunofluorescence of HPV+ tumor tissue revealed areas of cells co-expressing the identified markers of myofibril assembly, HPV surrogate marker p16, and stress-associated basal keratinocyte marker KRT17, indicating that the observed myocyte-like in vitro differentiation occurs in human tissue. We are the first to report that carcinoma cells can undergo a triggered myocyte-like differentiation, and our study suggests that the targeted differentiation of HPV+ HNSCCs might be therapeutically valuable.

## Background

Head and neck squamous cell carcinomas (HNSCC) are a diverse group of cancers originating from various head and neck regions, including the nasopharynx, oral cavity, oropharynx, hypopharynx, and larynx. Globally, HNSCC accounts for roughly 700,000 new cases and 380,000 deaths annually [1]. The 3- and 5-year overall survival rates for human papillomavirus (HPV) positive HNSCC patients are 80.0% and 75.0%, and for HPV-negative patients, 54.0% and 48.0% [2]. Due to this reason, it is imperative to develop new treatment strategies that specifically target tumors. The human papillomaviruses are a group of common viruses that infect skin and mucous membranes. They can cause warts, which usually dissolve without treatment, but certain types of HPV can cause cancer. There are over 100 types of HPV, which are classified into two groups: low-risk and high-risk (oncogenic). The low-risk HPVs, mostly HPV6 and 11, can cause harmless and temporary lesions. However, high-risk HPV infection may lead to malignant transformation. The high-risk group includes HPV16, 18, 31, 33, 35, 39, 45, 51, 52, 56, 58, 59, and 68 [3].

Differentiation therapy (DTH) is a therapeutic strategy that involves the utilization of diverse molecular agents capable of inducing differentiation in malignant cells. The rationale behind this approach is to promote the differentiation of cancer cells into specialized cell types, resulting in the removal of these cells from the proliferative compartment. This approach aims to promote terminal differentiation, which is regarded as a final branch of cellular development. Consequently, DTH has emerged as a promising treatment modality for cancer and has garnered significant attention in the scientific community [4].

A previous study discovered that HPV-negative HNSCC cell differentiation can be induced through cornification, leading to a loss of cell malignancy [5]. This finding introduced a new strategy and opportunity for targeted therapy in HPV-negative HNSCC. The successful reprogramming of cancer cells into differentiated cells has been implemented before, most notably in PML-RARα fusion-driven acute promyelocytic leukemia [6]. Nevertheless, it remains a challenge in other types of tumors, such as colon cancer and pancreatic ductal adenocarcinoma. The reason is that tumor-initiating cells (TICs) possess the ability to change their characteristics and functions, allowing tumors to avoid terminal differentiation through epigenetic mechanisms [7–9]. This phenomenon resembles the self-renewing properties of normal tissue regeneration [10]. Unfortunately, this characteristic might oppose the application of differentiation therapies in these types of cancer. However, our previous study found that differentiation therapy might represent an effective treatment strategy for HPV-negative HNSCCs. This is because terminal cornification, a non-reversible process, leads to programmed cell death and loss of malignant behavior. Squamous cell carcinomas arising from skin or mucosal tissue undergoing natural cornification appear vulnerable to this type of therapy, which must be investigated in further research.

The number of HPV+ immortalized HNSCC cell lines available is significantly limited to approximately eight lines [5, 11–13]. Developing primary cell cultures from naturally infected HPV-positive cancers is challenging [14]. However, we have managed to provide two such cell culture models. As of now, there is no existing documentation for a primary HPV-positive HNSCC differentiation model. Our system represents an opportunity to learn more about differentiation in this tumor type and may aid the identification of new therapy targets to target HPV-associated cancer types. This report provides an overview of the establishment and characterization of two in vitro HPV-associated HNSCC differentiation models and discusses their potential for cancer therapy.

## Materials and methods

### Human material and cell culture

Primary HPV+ HNSCC tissue was collected from patients who gave their informed consent during medically necessary surgeries. This was done in accordance with the declaration of Helsinki and was approved by the ethics committee of the Ruhr-University Bochum (AZ 2018-397_3), as described previously [14, 15]. In brief, between 0.1 and 1 g of tumor material was minced into <2 mm tissue pieces and digested using 5–10 mL of a 2.5 mg/mL Collagenase NB4 standard (Nordmark Biochemicals, Uetersen, Germany) solution in phosphate-buffered saline (PBS) + 3 mM CaCl_2_ for 1.5–2 h at 37 °C with gentle mixing every 5–10 min. Digested pieces and single cells were subsequently cultured in PneumaCult™-Ex Plus Medium, referred to as stem cell medium (SCM, #05041, Stemcell Technologies, Vancouver, Canada) supplemented as described [14]. Adherent cells were detached using Accutase (Capricorn Scientific, Ebsdorfergrund, Germany). The differentiation was performed using cardiac fibroblast medium (CFM, 316K-500, Cell Applications, San Diego, CA, United States) supplemented with 1% 200 mM L-Glutamine, 1% 100× penicillin/streptomycin, and 1% 250 µg/mL Amphotericin B solution (all three Capricorn Scientific). The cells were cultured in fresh medium twice a week.

### Mouse xenograft tumor models

Xenograft tumor tissue was derived from a previous investigation [15]. In that study, all experiments conducted on animals have been approved by the Institutional Animal Care and Use Committee of the University of Granada under procedures 12/07/2019/128. Such experiments have been carried out strictly with the European Convention for the Protection of Vertebrate Animals used for Experimental and Other Scientific Purposes (CETS #123) and the relevant Spanish law (R.D. 53/2013). The NSG mice (NOD.Cg-Prkdcscid Il2rgtm1Wjl/SzJ, The Jackson Laboratory, Bar Harbor, ME, United States) were housed in appropriate sterile filter-capped cages and fed and given water ad libitum, aged 5–6 weeks for xenografting of established cancer cell lines. During the xenograft experiments, blinding, randomization, and sample size estimation statistics were not performed.

### Histopathology analysis of HNSCC tissue

The paraffin-embedded tissue was sectioned with a 2 µm thickness using a Zeiss sliding microtome. Hematoxylin/Eosin (HE) staining was performed following standard protocols in a linear COT 20 tissue stainer by MEDITE, Burgdorf, Germany. The HE-stained sections were analyzed by a senior pathologist (F.B.), to establish a comparison between the patient characteristics of the original tumor and the xenograft tumor models. HPV diagnostics were performed by the Department of Pathology of Klinikum Bielefeld using a p16 staging protocol described previously [15]. In addition, HPV subtyping was performed using an HPV Easy-Typing Reverser Hybridization Kit and the HPV Easy Amplification Mix (AID GmbH, Straßberg, Germany).

### Cornification assay

The process of cornification was quantified using a pre-established protocol designed to isolate cornified envelopes [16]. In this experiment, we seeded triplicates of P1 and P2 cells into six-well plates using either SCM or differentiation medium until the cells reached a confluency of 80–90%. Following this, we washed the cells with PBS and detached them using Accutase from Capricorn Scientific, Ebsdorfergrund, Germany. Subsequently, we stained the cells with trypan blue from Sigma Aldrich and counted the number of live and dead cells using a Neubauer Chamber. Further, the cells were washed in PBS and subsequently mixed with 100 μL 4% SDS (Carl Roth GmbH, Karlsruhe, Germany) and 2% beta-Mercaptoethanol (Merck, Darmstadt, Germany) in PBS. The suspension was heated at 95 °C for 5–10 min. Finally, the number of cornified envelopes was counted using a Neubauer Chamber.

### Flow cytometry

Fresh single cells were collected from passages 3 (P2) and 5 (P1) and suspended in PBS. Differentiation medium-treated cells were measured after 72 h using Annexin-PI staining (Annexin V-FITC Kit, Miltenyi Biotec, Gladbach, Germany) to measure apoptosis and death rates upon triggered differentiation. Three technical replicates were used (*n* = 3). The cell staining was processed according to the manufacturer’s standard protocol. The measurement was performed using a Miltenyi MaxQuant16 flow cytometer.

### Cell viability

Cell viability was assessed for 7 days in SCM and differentiation medium cultures using the CellTiter 96(R) AQueous One 3 Solution Assay (Promega, Madison, Wisconsin, USA). The absorbance at 490 nm was recorded using the TECAN Infinite 200 (Tecan, Männedorf, Switzerland).

### Proliferation assay

In this study, three independent experiments were conducted to investigate the growth characteristics of P1 and P2 cells. Initially, 5 × 10^5^ cells were seeded into T-75 plates in a serum-containing medium (SCM). Upon reaching 70–90% confluency, the cells were washed PBS and detached using Accutase. The cells were manually counted using a Neubauer Chamber and subsequently washed in PBS, resuspended in a culture medium, and re-seeded using a dilution factor of 1–12. Growth curves were generated over a period of 4–20 passages, depending on the proliferation characteristics of each cell culture.

### Wound healing

To generate a confluent cell monolayer, individual cells were seeded into a six-well titer plate and grown using SCM and differentiation medium for 8 days. Once the cells reached a confluent state, a gap was introduced into the monolayer by mechanical scratching. The wound healing properties were then evaluated for a total of 96 h.

### Indirect immunofluorescence of HNSCC cells and tissue

Indirect Immunofluorescence (IF) of HNSCC cell cultures was performed as described previously [17]. Tumor tissue of patients and xenograft tumor models were analyzed by IF as established in prior studies [8]. Samples were imaged using an LSM780 confocal microscope (Zeiss, Oberkochen, Germany) and ZEN software (Zeiss). DNA was stained with DAPI (Sigma Aldrich, St. Louis, MO, United States) using 2 µg/ml diluted in PBS + 0.1% bovine serum albumin (Capricorn Scientific). Primary antibodies: rabbit-anti-human ACTA1 (1:200, EPR16769, ab179467, Abcam, Cambridge, UK), rabbit-anti-human CDKN2A/p16INK4a (EPR1473; 1:200; Abcam), rabbit-anti-human EpCam (EGP40/1556R; 1:100; Novus Biologicals, Centennial, CO, United States), mouse HPV antibody cocktail (1:100, CAMVR-1 & C1P5, Z2657MS, Thermo Fisher Scientific, Waltham, MA, United States), rabbit-anti-human Ki67 (1:200, SP6, ab16667, Abcam), mouse anti-human Tropomyosin (1:100, F-6, sc-74480, Santa Cruz Biotechnology, Dallas, TX, United States). Secondary antibodies: goat-anti-mouse-IgG-Alexa Fluor-555 (1:400, A21422), donkey-anti-rabbit-IgG-Alexa Fluor-488 (1:400, A11008), and goat-anti-guinea pig-IgG-Alexa Fluor-647 (1:400, A21450, all Thermo Fisher Scientific, Waltham, MA, United States). The actin cytoskeleton was stained using phalloidin-PF647 for 30 min (1:50, Promokine, Heidelberg, Germany).

### RNA isolation

Total RNA was isolated from cell culture (*n* = 3) using innuPREP DNA/RNA Mini Kit (Analytik Jena, Jena, Germany) according to the manufacturer’s instructions. RNA concentration was determined with a BioDrop Duo+ spectral photometer (Biochrom, Holliston, MA, USA).

### Analysis of RNA-Seq data

RNA-Seq analysis was performed as previously described [5].

## Results

### Establishment of a primary HPV-positive HNSCC cell culture model

**Fig. 1:**
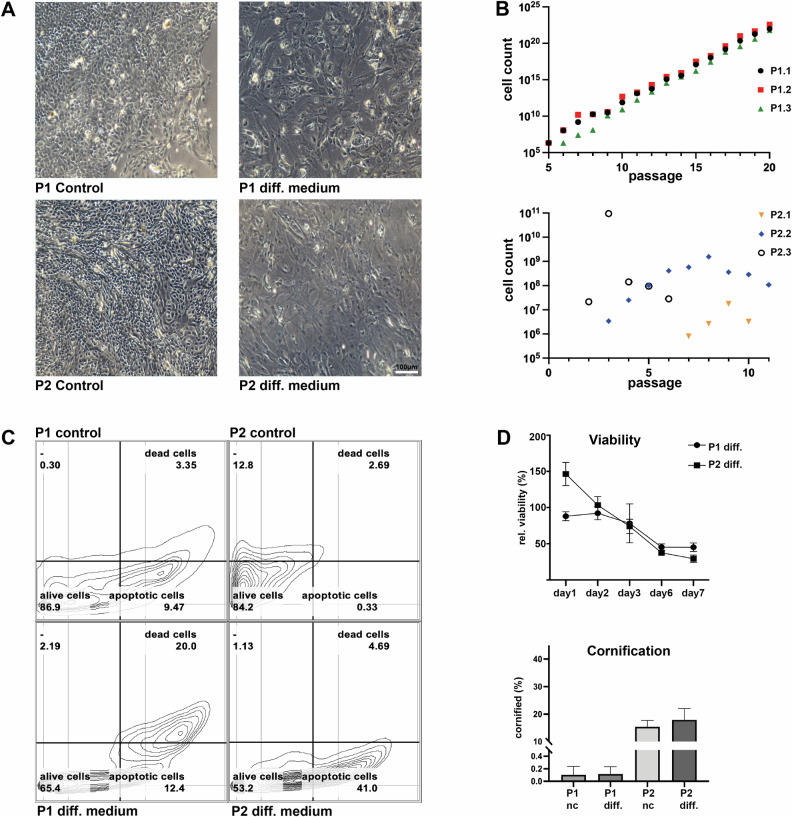
HPV+ HNSCC differentiation model reveals loss of cell viability. **A** Cultures of P1 and P2 display adherent monolayer cells in SCM with epithelial morphology (undifferentiated). Treatment of SCM cultures with differentiation medium results in the loss of epithelial morphology and enlarged cells with irregular shapes. **B** Graphs show exponential proliferation of P1 cells for 20 passages (5 months), whereas cells of P2 stop proliferation within 11 passages; 3 independent experiments for each patient are displayed. **C** Apoptosis and death rate of differentiation medium-treated cells increase within 72 h; measured by Annexin-PI staining. Three technical replicates were used (*n* = 3). **D** Differentiation medium treatment for 7 days reduced cell viability. Differentiation medium treatment does not increase cornification in cultures of P1 and P2. Cornification was very strong in P2 cells in both media, whereas P1 explants showed low cornification levels.

We conducted a study to examine the effect of a potential differentiation treatment approach for HPV-positive HNSCCs. For this purpose, we established two primary cell culture models derived from oropharyngeal HNSCC of two male patients aged 49 and 81 (P1 and P2, respectively) who had tested positive for human papillomavirus 16 (Fig. 1A) using standard diagnostic tests. Cells of P1 were derived from a previously established xenograft tumor model [15], while P2 was directly established from the patient’s tissue. The xenograft tumors of P1 closely resembled the original tumor in histology [15], and we isolated cells from the xenograft tumor tissue that could be expanded for more than 20 passages in stem cell medium (SCM) without phenotypic changes (Fig. 1B). The cells obtained from P2 could be cultured in SCM for 6 to 11 passages (Fig. 1B).

### Triggered differentiation of HPV-positive HNSCC cells activates myocyte-like gene expression

To induce the differentiation of HPV+ HNSCC cells, we treated the cells of P1 and P2 with a differentiation medium called cardiac fibroblast medium (CFM), previously found to trigger differentiation in HPV-negative HNSCC cells [5]. Differentiation medium treatment resulted in an enlarged irregular cell morphology (Fig. 1A). After 72 h, the proportion of apoptotic cells within P1 increased from 9.47% to 12.4%, and death rates were elevated from 3.35% to 20%. Meanwhile, in cells from P2, apoptotic cells increased from 0.33% up to 41.0%, and dead cells increased from 2.69% to 4.69% (Fig. 1C). During the experiment, the cell viability was assessed for seven days. The results show a reduction of about 50% when compared to the SCM cultivation (Fig. 1D). The cornification of HPV-positive HNSCC cells was unaffected by the differentiation treatment (Fig. 1D), and there was no increase in migration (suppl. Figure S1).

**Fig. 2:**
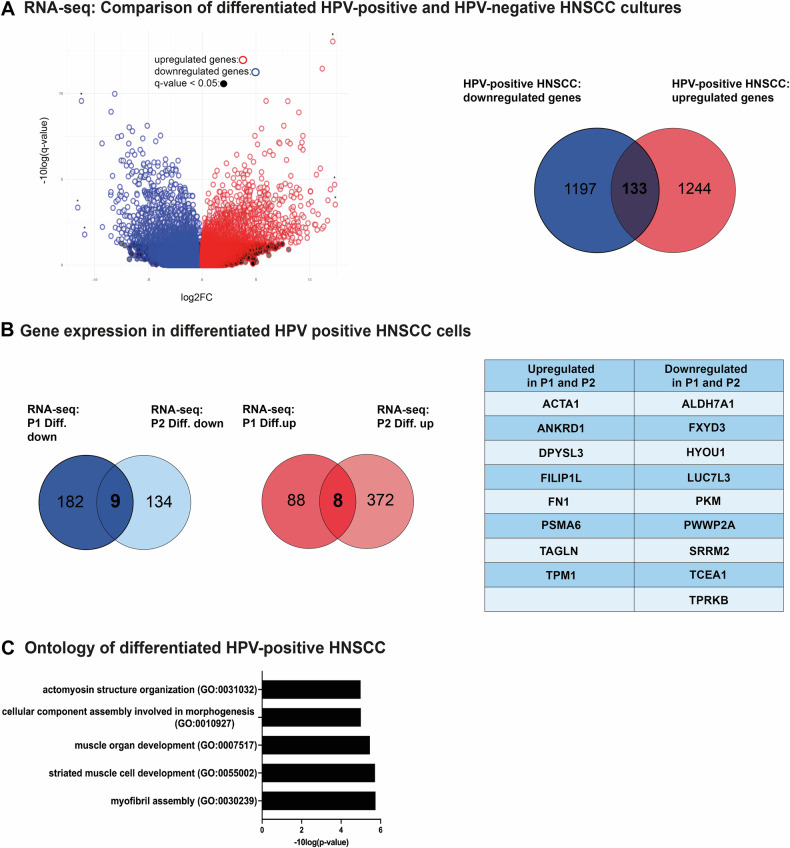
HPV-positive HNSCC cells differentiate into myocyte-like cells. **A** The global expression data highlights the contrasting characteristics of HPV-positive and HPV-negative HNSCC differentiation, indicating different mechanisms of differentiation. **B** Gene expression analysis of P1 and P2 cell cultures shows genes upregulated or downregulated upon differentiation. **C** Analysis of nine genes upregulated in differentiated cells of both patients revealed five significant Gene Ontology Biological Process pathways associated with HPV+ HNSCC cell differentiation. Analysis of downregulated genes gave no results. Three technical replicates were used (*n* = 3) with a statistical cut-off *p* value of 0.05.

**Fig. 3:**
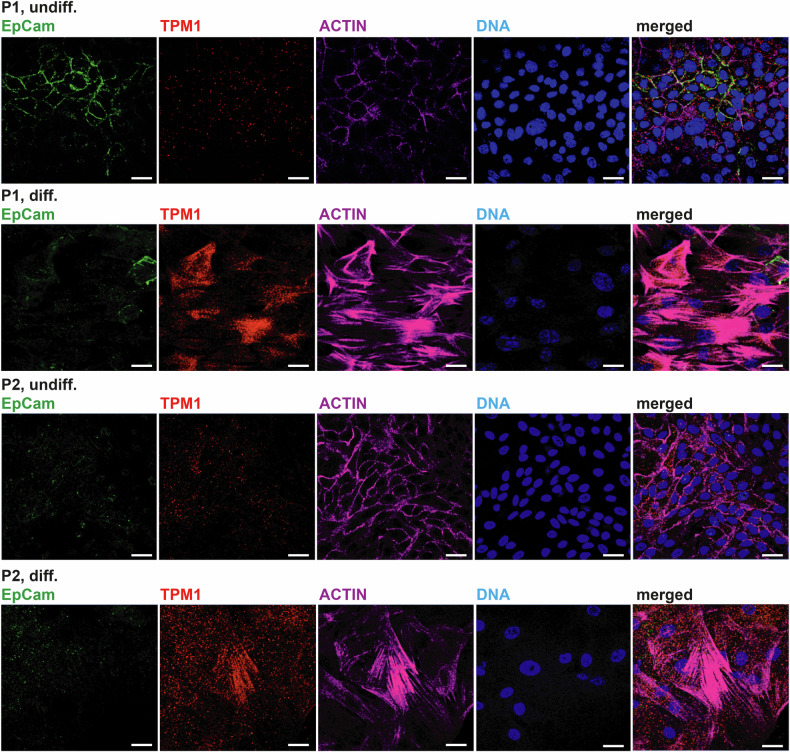
Differentiated HPV+ HNSCC cell cultures express markers of myocyte differentiation. When treated with differentiation medium, P1 cells lose expression of EpCam, whereas cells of P2 are negative for EpCam in any medium. Both cell cultures upregulate muscle cell marker tropomyosin 1 (TPM1) and show a reorganization of the actin cytoskeleton from cortical actin to the formation of parallel fibers, reminiscent of muscle lineage differentiation; scale bars = 10 µm.

RNA-Seq was conducted to understand the signaling processes leading to a loss of cell fitness in our primary cell cultures upon differentiation. We compared the impact of the differentiation medium on the expression of HPV-negative cells from a recent study and our HPV-positive cell cultures (Fig. 2A). Our findings suggest that the differentiation medium affects both HNSCC subtypes, but the underlying mechanisms differ. Our data show 1244 differentially upregulated and 1197 downregulated genes (Fig. 2A). Genes commonly upregulated upon differentiation of both HPV-positive cell cultures were *ACTA1*, *ANKRD1*, *FILIPIL*, *FN1*, *PSMA6*, *TAGLN*, and *ТРМ1* (Fig. 2B). Gene Ontology analysis of these genes revealed the activation of a myocyte-like expression pattern. Significantly enriched gene sets that differentiated cells of both patients have in common include actomyosin structure organization, cellular component assembly involved in morphogenesis, muscle organ development, striated muscle cell development, and myofibril assembly (Fig. 2C). The genes downregulated in both patients were *ALDH7A1*, *FXYD3*, *HYOU1*, *LUC7L3*, *PKM*, *PWWP2A*, *SRRM2*, *TCEA1*, and *TPRKB* (Fig. 2B) with no significantly enriched gene sets. Differentiated cells of both patients displayed changes in their actin filament structure, shifting from a cortical distribution to a parallel fiber arrangement that resembled a myocyte phenotype. This was indicated by a strong TPM1 protein signal in indirect immunofluorescence staining (IF) aligned with the parallel actin filaments indicative of muscle-lineage cells (Fig. 3).

In contrast, undifferentiated cells grown in the SCM medium exhibited no detectable TPM1 expression. SCM and differentiation medium-cultured cells of P1 and P2 expressed HPV-related proteins, but differentiation treatment strikingly diminished Ki67 proliferation marker expression (suppl. Figure S2). Notably, HPV-related proteins were detected by IF under both conditions.

### Identified myocyte-associated markers are expressed in HPV-positive HNSCC tissue

**Fig. 4:**
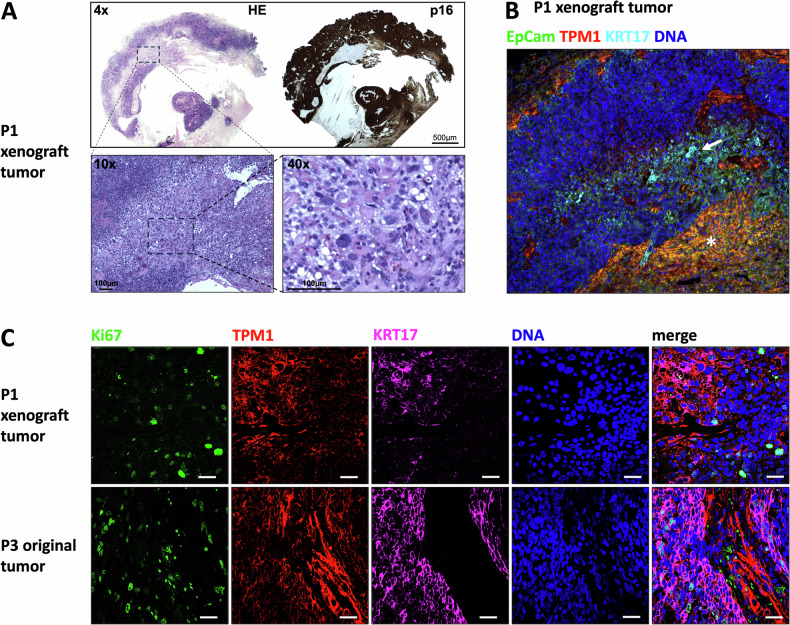
Myocyte-like differentiation occurs in HPV+ HNSCC tissue. **A** Xenograft tumor of P1 with cystic/necrotic core. Hematoxylin/eosin (HE) and p16 staining of the same paraffin block were shifted into a similar orientation for comparison. **B** IF staining of P1 xenograft tumor reveals cells co-expressing TPM1 and KRT17 (arrow) and tissue positive for TPM1 and EpCam (star); 10x magnification. **C** P1 xenograft tumor and P3 original tumor show Ki67 in areas with lower TPM1/KRT17 expression; scale bars = 10 µm.

To examine the presence of myocyte markers in human tumors, an IF staining was performed to detect TPM1, p16, and KRT17. KRT17 is a stress-keratin and an early differentiation marker in HPV-HNSCCs [5]. The tumor tissue of three patients (P1, P2, and P3) and the xenograft tumor tissue of P1 from a previous study were examined. The IF revealed the abundance of cells expressing the above-mentioned myocyte markers in the tumor tissues of the selected patients. ACTA1 was found to be expressed in cells staining co-positive for HPV-related proteins (Suppl. Fig. S3). In addition, we observed strong TPM1 expression in desmoplastic stromal areas and in p16/KRT17+ cells with epithelial morphology (Suppl. Fig. S4). Thus, a subpopulation of HPV+ tumor cells expresses myocyte markers. In the original tumor specimens of P1 and P2, these cells were diffusely spread throughout the tumor tissue. HNSCC xenograft tumors are known to display a lymph node metastasis-like cystic/necrotic growth pattern [5, 18]. Xenograft tumor tissue of P1 contained enlarged cells with pleomorphic nuclei towards the core of the inner cyst (Fig. 4A). These stained strongly positive for KRT17 (Fig. 4B). By confocal microscopy imaging of this area, we observed a correlation between TPM1 and KRT17 expression and Ki67 proliferating cells located in the TMP1-/KRT17- population (Fig. 4C). This was also detected in the original tumor tissue of P3. Our data indicate that myocyte markers and KRT17 represent markers of differentiated non-proliferative HPV+ HNSCC cells.

## Discussion

This study successfully established two primary cell culture models of HPV-associated HNSCC. The use of primary cell cultures was imperative due to the unfavorable genetic or epigenetic changes associated with the immortalization process of cell lines [19]. Thus, immortalized cell lines are not an adequate substitute for tumor differentiation studies [20]. However, it is known from previous studies that benign cell types, for example, fibroblast-like cells derived from the desmoplastic stroma, can contaminate primary carcinoma cell cultures [21]. We employed two primary models derived from xenograft tumor tissue (P1) or directly from patient material (P2). Both showed epithelial morphology and expression of the epithelial marker EpCam (P1) or signs of epithelial differentiation by cornification (P2). Moreover, both cultures expressed HPV-related proteins that were not abrogated by the induced myocyte-like differentiation. Combined expression of markers of myocytes and HPV was also found in human HPV+ HNSCC tissue. Thus, we conclude that our primary model system faithfully recapitulates the mechanisms of human HPV+ HNSCC differentiation.

Moreover, our findings demonstrate that we can force HPV-positive HNSCC cells to differentiate into a robust myocyte phenotype. During this differentiation, we observed a morphological structural reorganization from an epithelial to a myocytic phenotype, indicating a successful trans-differentiation. We accounted for this differentiation phenotype by demonstrating that markers like *TPM1*, *TAGLN*, and *ACTA1* were upregulated in both culture models. In vertebrates, TPM1 plays a crucial role in regulating striated muscle contraction dependent on calcium [22]. TAGLN is a protein highly present in smooth muscle cells and is commonly used as a standard marker for these cells [23]. RNA-Seq revealed that actomyosin structure organization, muscle organ development, striated muscle cell differentiation, and myofibril assembly are the mechanisms behind the differentiation process. Further, muscle markers TPM1/ACTA1 and the recently described HNSCC differentiation marker KRT17 [5] correlate in the cell cultures and the original tumor tissue, indicating that our differentiation model faithfully reflects the differentiation behavior in human tumors.

Our previous study showed that in HPV-negative HNSCCs, terminal differentiation resulted in cornification, accompanied by the epigenetic loss of cell malignancy [5]. In contrast, our findings here indicate that HPV-positive HNSCC cells undergo a myocyte-like differentiation, which also causes a loss of malignant characteristics. Thus, different phenotypes were triggered by the same differentiation protocol in dependence on the HPV status.

Furthermore, HPV+ HNSCC cells that have undergone induced differentiation exhibit signs of epithelial-to-mesenchymal transition (EMT), which is linked to metastatic spread and represents a hallmark of cancer [24]. Notably, in this model, the EMT-like transition leads to a less aggressive phenotype, resulting in reduced proliferation and viability, decreased Ki67 expression, and increased cell death, but induced no changes in migration. Regarding EMT as a cancer hallmark, recent reports demonstrate that efficient invasion and metastasis require the expression of *ZEB1* and *SNAIL* [25, 26]. The expression data from both of our patient-derived models did not show any evidence of such invasive or malignant signaling.

The upregulation of Filamin A Interacting Protein 1 Like (*FILIP1L*) and the downregulation of *ALDH7A1* induced here could be responsible for the loss of malignant characteristics. *FILIP1L* is described to induce loss of proliferation and migration in endothelial cells and is known to inhibit melanoma growth when expressed in tumour-associated vasculature [27]. *ALDH7A1* is generally known as a stemness marker in prostate cancer [28] and is involved in the growth of pancreatic ductal adenocarcinoma [29] and might thus be implicated in the maintenance of the undifferentiated phenotype of long-term repopulating HPV+ HNSCC cells. Further studies should investigate the role of these effectors, which are highlighted here.

The successful reprogramming of cancer cells into differentiated cells has been implemented in other malignancies, most notably PML-RARα fusion-driven acute promyelocytic leukemia [6]. Besides, in the field of sarcoma, researchers at Cold Spring Harbor Laboratory have found a promising DTH approach by targeting NF-Y with CRISPR. The group differentiated rhabdomyosarcoma cells into muscle cells, representing that tissue’s normal differentiation path [30]. To our knowledge, our team is the first to present an induced morphological alteration from cancer cells into muscle-like cells in carcinomas [5]. Both approaches differ in their experimental implementation but lead to similar results. According to our findings, the targeted induction of myogenic differentiation in tumor cells confers therapeutic benefits not only in sarcomas but also in carcinomas.

Most importantly, a clinical approach based on our data must be established to test the suitability of our targeted differentiation for therapeutic intervention against HNSCCs. Although an in vitro differentiation protocol has been developed successfully here, its translation into a therapeutic drug regimen will be challenging and needs to be pursued in follow-up studies. Further research is necessary to identify an alternative composition of mediators that can mimic the impact of the differentiation medium. Once a more practical treatment method is found, testing its translational value in xenograft tumor models of HNSCCs in immunodeficient mice is essential. This method of treatment may also be useful for other HPV-associated cancers. Overall, this system could be a versatile tool for explicit pharmacological studies, leading to a more comprehensive understanding of HPV-positive HNSCC in general.

### Supplementary information

Supp Material

Supp Tables

## Data Availability

All RNA sequencing raw data are available on the NCBI Gene Expression Omnibus database under the GEO accession number GSE271483.
