# Supplementary material for: Human papillomavirus-associated head and neck squamous cell carcinoma cells lose viability during triggered myocyte lineage differentiation
Source: Cell Death Dis. 2024 Jul 19;15(7):517. doi: 10.1038/s41419-024-06867-4 (PMC11271587; doi:10.1038/s41419-024-06867-4)
Supplement: Supplementary file 1 — Supp Material [file 41419_2024_6867_MOESM1_ESM.docx]

**SUPPLEMENTARY MATERIAL**

**Supplementary Figure S1: Wound healing assay of HPV+ differentiation models.**

**Supplementary Figure S2: HPV+ HNSCC cell differentiation induces a loss of Ki67 expression.**

**Supplementary Figure S3: HPV+ cells in HNSCC tissue express muscle markers.**

**Supplementary Figure S4: Myocyte-like HPV+ cells in HNSCC tissue display signs of differentiation.**

The following supplementary figures can be found in a separate tabular file.

**Supplementary Table S1: Patient characteristics**

**Supplementary Table S2: The differential expression analysis of the RNA-seq of patient 1: diff. medium upregulated**

**Supplementary Table S3: The differential expression analysis of the RNA-seq of patient 1: diff. medium downregulated.**

**Supplementary Table S4: The differential expression analysis of the RNA-seq of patient 2: diff. medium upregulated**

**Supplementary Table S5: The differential expression analysis of the RNA-seq of patient 2: diff. medium downregulated.**

**Supplementary Figures**

**
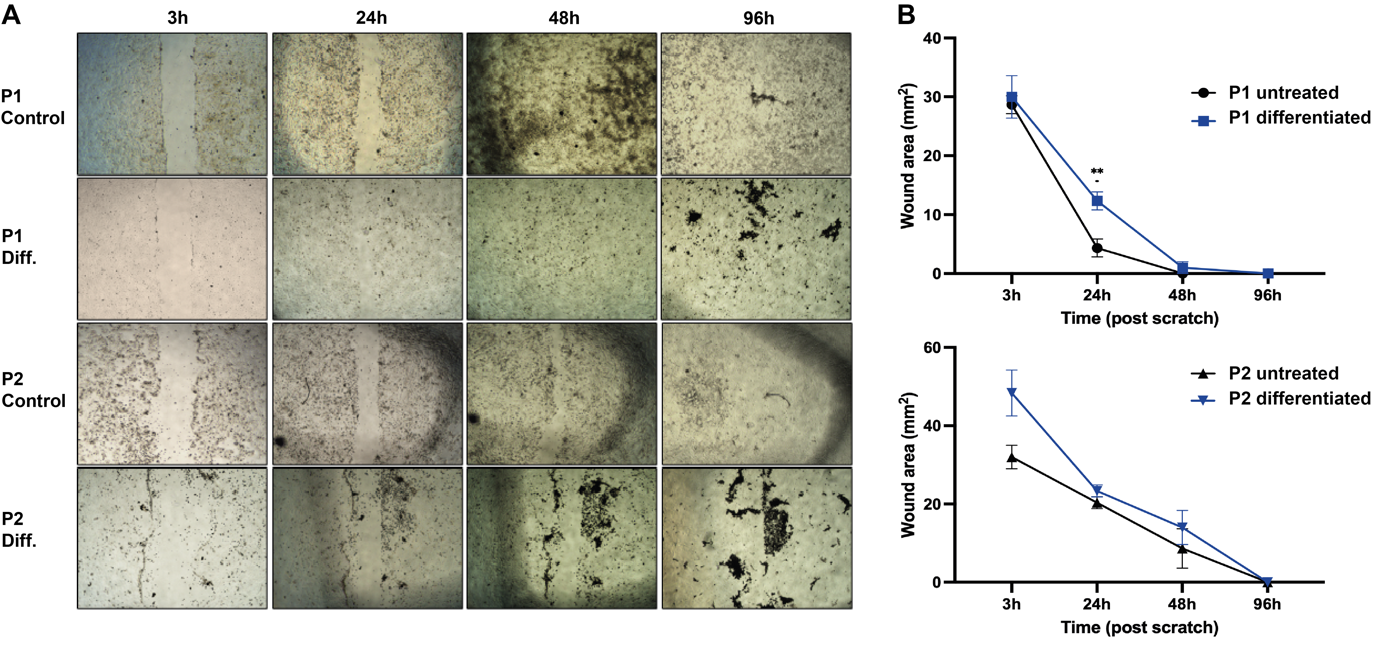
**

**Supplementary Figure S1: Wound healing assay of HPV+ differentiation models. (A)** When treated with the differentiation medium, P1 cells significantly lose wound healing attributes (p-value=0.03), whereas cells of P2 did not change. **(B)** Wound healing area after scratch assay of P1 and P2 undifferentiated and differentiated cells; scale bars = 10 µm.

**
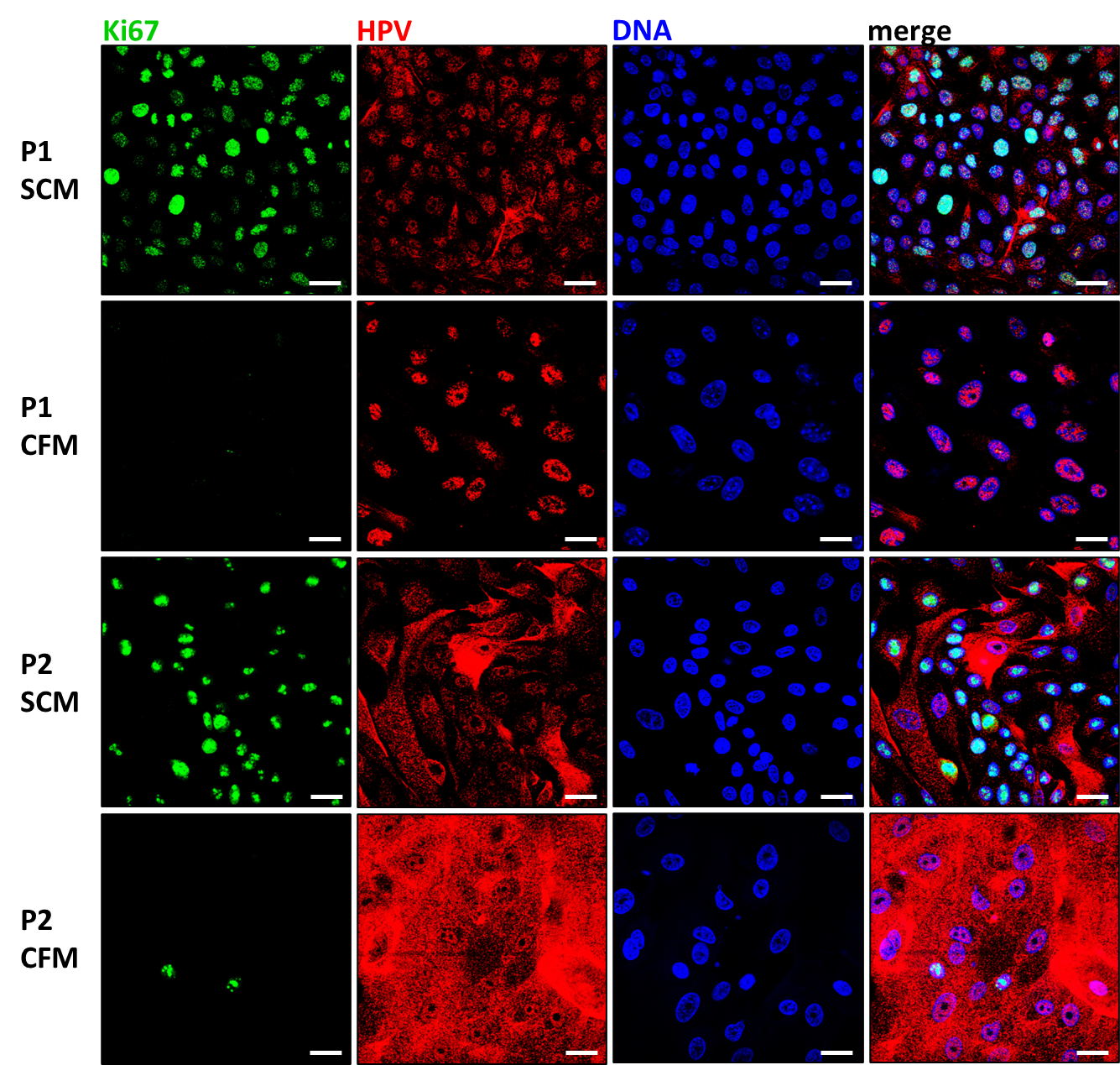
**

**Supplementary Figure S2: HPV+ HNSCC cell differentiation induces a loss of Ki67 expression.** Cells of P1 and P2 lose Ki67 proliferation marker expression when cultured in differentiation medium (CFM) instead of stem cell medium (SCM). HPV-related proteins are expressed under both conditions; scale bars = 10µm.

**
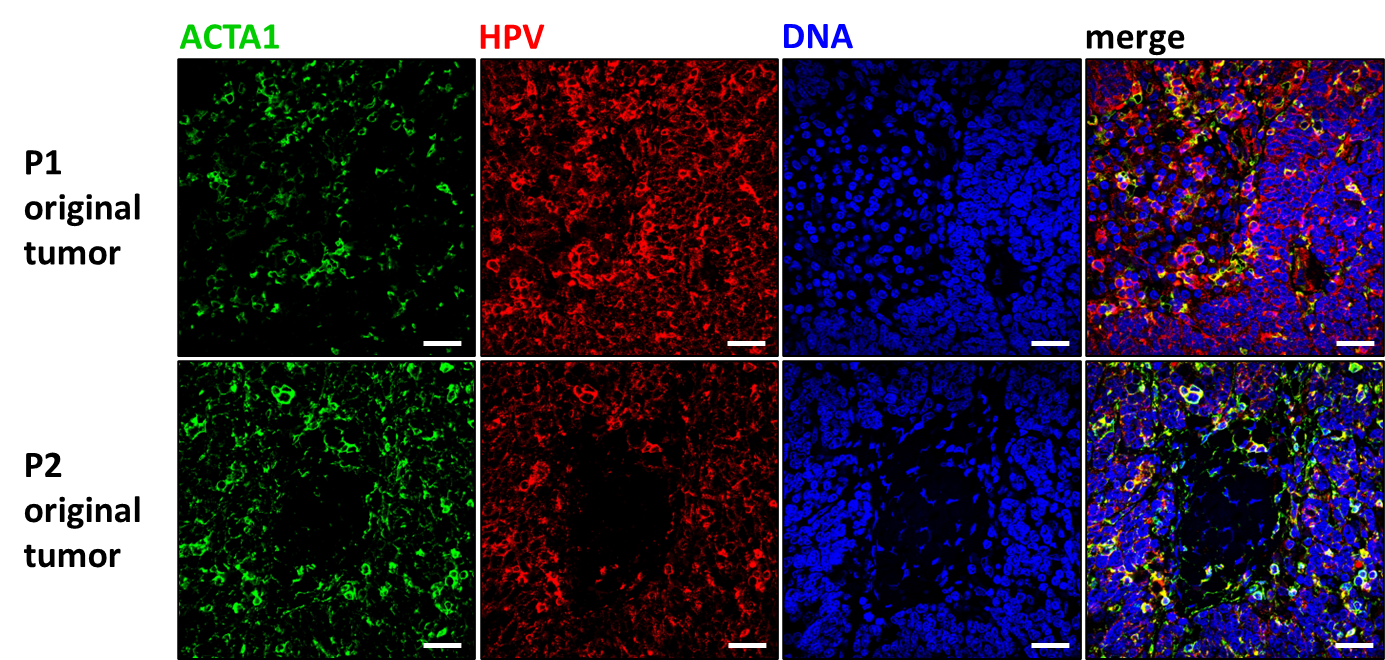
**

**Supplementary Figure S3: HPV+ cells in HNSCC tissue express muscle markers.** Myocyte lineage protein ACTA1 stains co-positive with HPV-related proteins in human HNSCC tissue of P1 and P2; scale bars = 10µm.

**
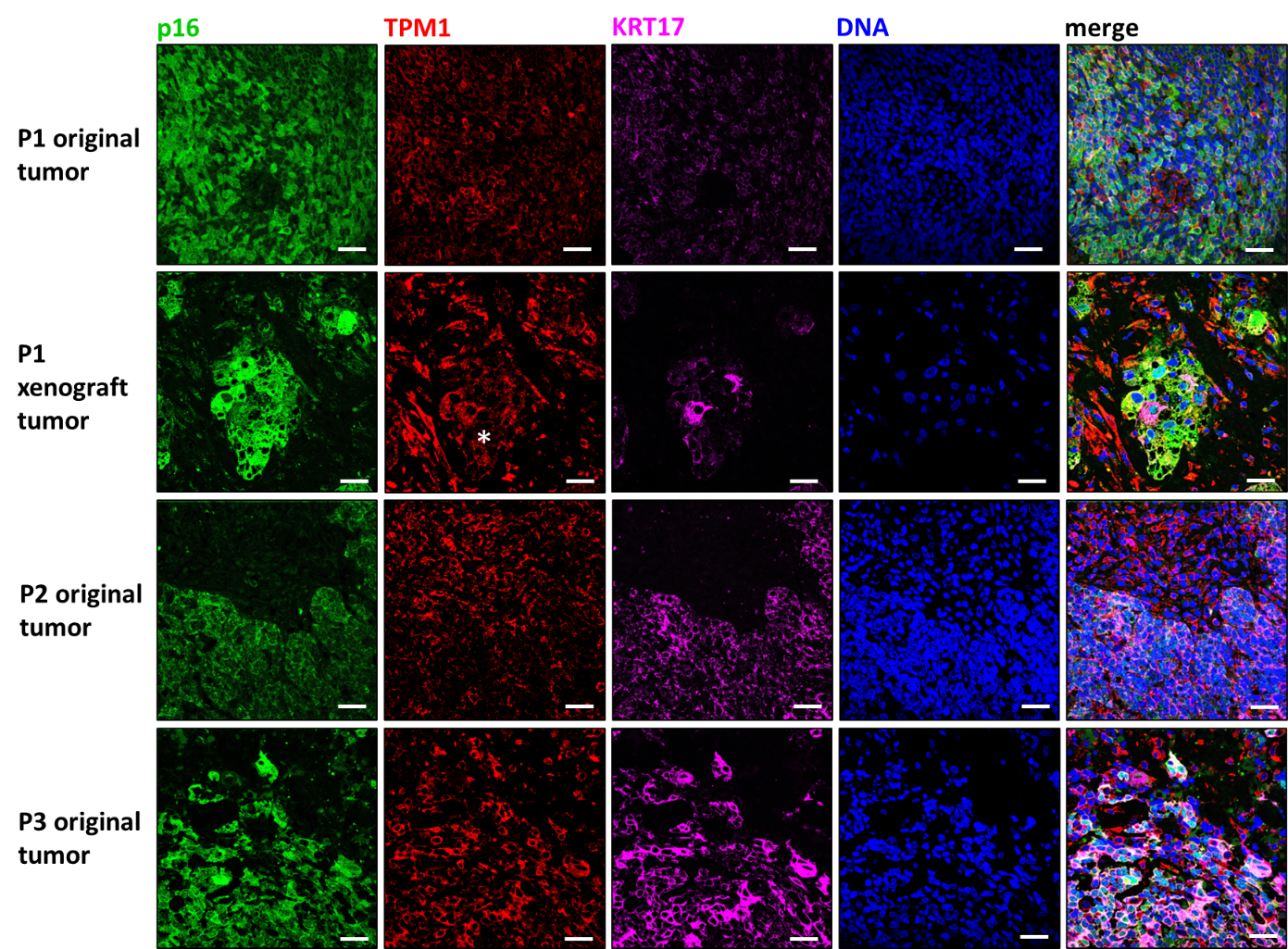
**

**Supplementary Figure S4: Myocyte-like HPV+ cells in HNSCC tissue display signs of differentiation.** Cells in tumors of P1-P3 show simultaneous expression of HPV surrogate marker p16, myocyte lineage marker TPM1, and differentiation marker KRT17 (Quelle); scale bars = 10µm.
